# C2 translaminar screw fixation in pediatric occipitocervical fusion

**DOI:** 10.1007/s00381-022-05471-1

**Published:** 2022-04-14

**Authors:** Young M. Lee, Alex Y. Lu, Taemin Oh, Joan Y. Hwang, Daniel C. Lu, Peter P. Sun

**Affiliations:** 1 Department of Neurological Surgery, University of California, San Francisco, CA USA; 2 Department of Neurological Surgery, David Geffen School of Medicine, University of California, Los Angeles, CA USA; 3 Department of Neurological Surgery, University of California San Francisco Benioff Children’s Hospital Oakland, CA Oakland, USA

**Keywords:** Occipitocervical instability, Translaminar screw, Pediatric spine, Spinal fusion, Atlantoaxial fixation

## Abstract

**Purpose:**

Rigid occipitocervical (O-C) instrumentation can reduce the anterior pathology and has a high fusion rate in children with craniovertebral instability. Typically, axis (C2) screw fixation utilizes C1–C2 transarticular screws or C2 pars screws. However, anatomic variation may preclude these screw types due to the size of fixation elements or by placing the vertebral artery at risk for injury. Pediatric C2 translaminar screw fixation has low risk of vertebral artery injury and may be used when the anatomy is otherwise unsuitable for C1–C2 transarticular screws or C2 pars screws.

**Methods:**

We retrospectively reviewed a neurosurgical database at UCSF Benioff Children’s Hospital Oakland for patients who had undergone a cervical spinal fusion that utilized translaminar screws for occipitocervical instrumentation between 2002 and 2020. We then reviewed the operative records to determine the parameters of C2 screw fixations performed. Demographic and all other relevant clinical data were then recorded.

**Results:**

Twenty-five patients ranging from 2 to 18 years of age underwent O-C fusion, with a total of 43 translaminar screws at C2 placed. Twenty-three patients were fused (92%) after initial surgery with a mean follow-up of 43 months. Two patients, both with Down syndrome, had a nonunion. Another 2 patients had a superficial wound dehiscence that required wound revision. One patient died of unknown cause 7 months after surgery. One patient developed an adjacent-level kyphosis.

**Conclusion:**

When performing occipitocervical instrumentation in the pediatric population, C2 translaminar screw fixation is an effective option to other methods of C2 screw fixation dependent on anatomic feasibility.

## Introduction

Occipitocervical (O-C) fusion is required for a variety of childhood craniocervical abnormalities that create overt instability or significant anterior compressive pathology. Rigid O-C constructs with screw fixation at C2 have superior biomechanical profiles and are highly likely to fuse. Additionally, they are able to reduce anterior pathology [1–7]. Screw fixation of C2 with C1–C2 transarticular and C2 pars screw has been expertly utilized in children for O-C fusion [8–10]. However, in addition to the inherent risk of vertebral artery injury [11], placement of C1–C2 transarticular and C2 pars screw can be complicated in children whose anatomy can be too small for commercially available screws or unsuitable because of an aberrant course of the vertebral artery in congenital and developmental craniocervical abnormalities.

C2 fixation with translaminar screws was first described by Wright in both adult and pediatric patients [11–13]. Consequently, the anatomy of C2 in children can be suited for translaminar screws unilaterally or bilaterally even when C2 pars screws or transarticular screws are not a feasible option. However, C2 translaminar screw placement can be challenging in younger children with smaller sized C2, and the midline hardware can reduce an already limited surface area for fusion. We report the use of C2 translaminar screw fixation for O-C fusion in a series of 25 pediatric children with long-term follow-up.

## Methods

Consecutive patients ages 18 years or younger who underwent O-C fusion incorporating C2 translaminar screw fixation at UCSF Benioff Children’s Hospital Oakland between 2002 and 2020 were identified. Patients who underwent O-C fusion with alternative C2 fixation techniques were not included in the study. Demographics and all other relevant patient data were then recorded.

**Fig. 1:**
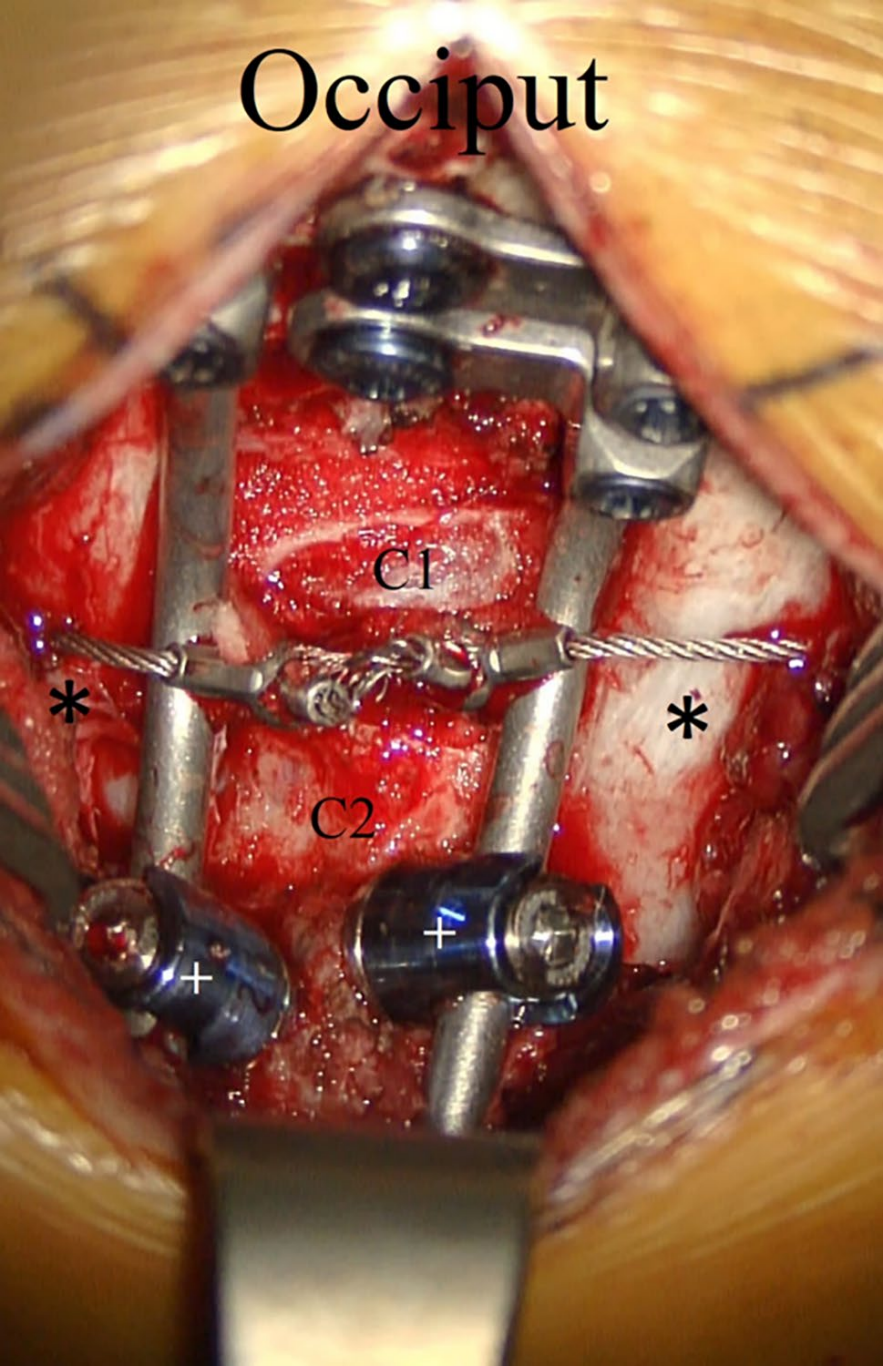
Intraoperative anatomy of bilateral C2 translaminar screw fixation. Translaminar screws ( +) have been inserted into C2 without offset connectors for fixation to the occiput. Rib autograft (*) is applied bilaterally and secured in place with Songer wiring. Decortication is performed over the occiput, the posterior ring of C1, and the laminae of C2

All patients underwent preoperative thin-cut CT with coronal and sagittal reconstructions and MRIs. All patients had plate-rod or rod constructs with rigid occipital screw fixation and 3.5 mm translaminar polyaxial screw fixation (Fig. 1). The minimum thickness of the lamina considered for the 3.5 mm diameter screws was 4 mm. Serial lateral, neutral, flexion, and extension plain films were obtained postoperatively. In those patients where fusion could not be documented on plain films, postoperative CT scans were obtained.

### Surgical technique

Patients were placed prone in either cervical collar or Halo immobilization. Patients with severe instability and cord signal change or severe spinal cord compression were flipped prone in a Halo ring and vest in order to maintain the head and cervical spine in a stable neutral position. The cervical collar or posterior vest was then removed, and the patient was attached to the head holder with Mayfield pins or Halo adapter. Appropriate alignment was verified with lateral fluoroscopy. Pre- and post-flip motor-evoked and somatosensory-evoked potentials were obtained and monitored throughout the duration of the operation. The occiput, craniocervical junction, and posterior upper cervical spine was exposed in standard fashion.

**Fig. 2:**
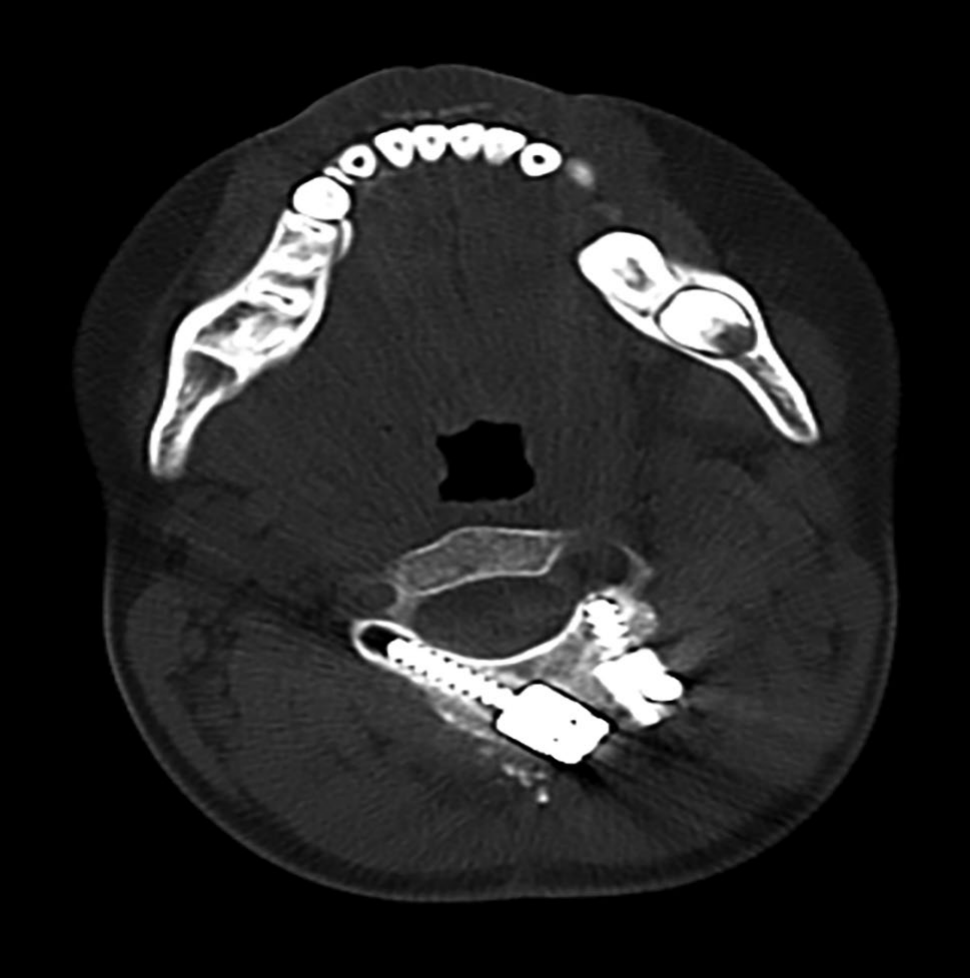
Translaminar C2 placement in patients with small C2 spinous process anatomy. An axial CT scan at the level of C2 is shown in a patient with a small C2 spinous process requiring placement of a notably shorter length left ipsilateral translaminar screw in a more ventral direction and lateral position, contralateral to a standard length right-sided translaminar screw in standard trajectory

For C2 translaminar screw fixation in older children with adequately sized C2, the surgical technique for entry and trajectory was performed as described by Wright [12, 13]. For younger children with small lamina, the technique differed for bilateral screw insertion. The smaller C2 spinous process in children sometimes did not allow for vertically crossed contralateral translaminar screws. In these cases, the spinous process was partially removed, and entry points were then chosen at the dorsal aspect of each lamina at a point that allowed for 2 entries into each ipsilateral lamina (Fig. 2). The laminae were then probed using a small pedicle finder or hand drilled (Medtronic Sofamor Danek, TN) following the dorsal downslope of the lamina. For smaller laminae where the screw threads engaged the inner cortex of the bone, tapping was needed to desired length. A small ball-tipped probe was used to palpate the hole in order to verify that there was no breach of cortex into the spinal canal. An appropriate-length 3.5 mm diameter polyaxial screw was inserted along the same trajectory. If there was sufficient room on C2, a contralateral screw was inserted in the same manner. However, crowding at the polyaxial screw heads could limit the trajectory of the second screw into a more ventral direction, and in this situation, the length had to be shortened appropriately (Fig. 2). The polyaxial screw heads were then connected to the rod or rod plate with offset connectors (Vertex, Sofamor Danek Medtronic, TN) needed in some patients. The reduction of the anterior pathology was performed by head positioning and/or instrumentation manipulation.

**Fig. 3:**
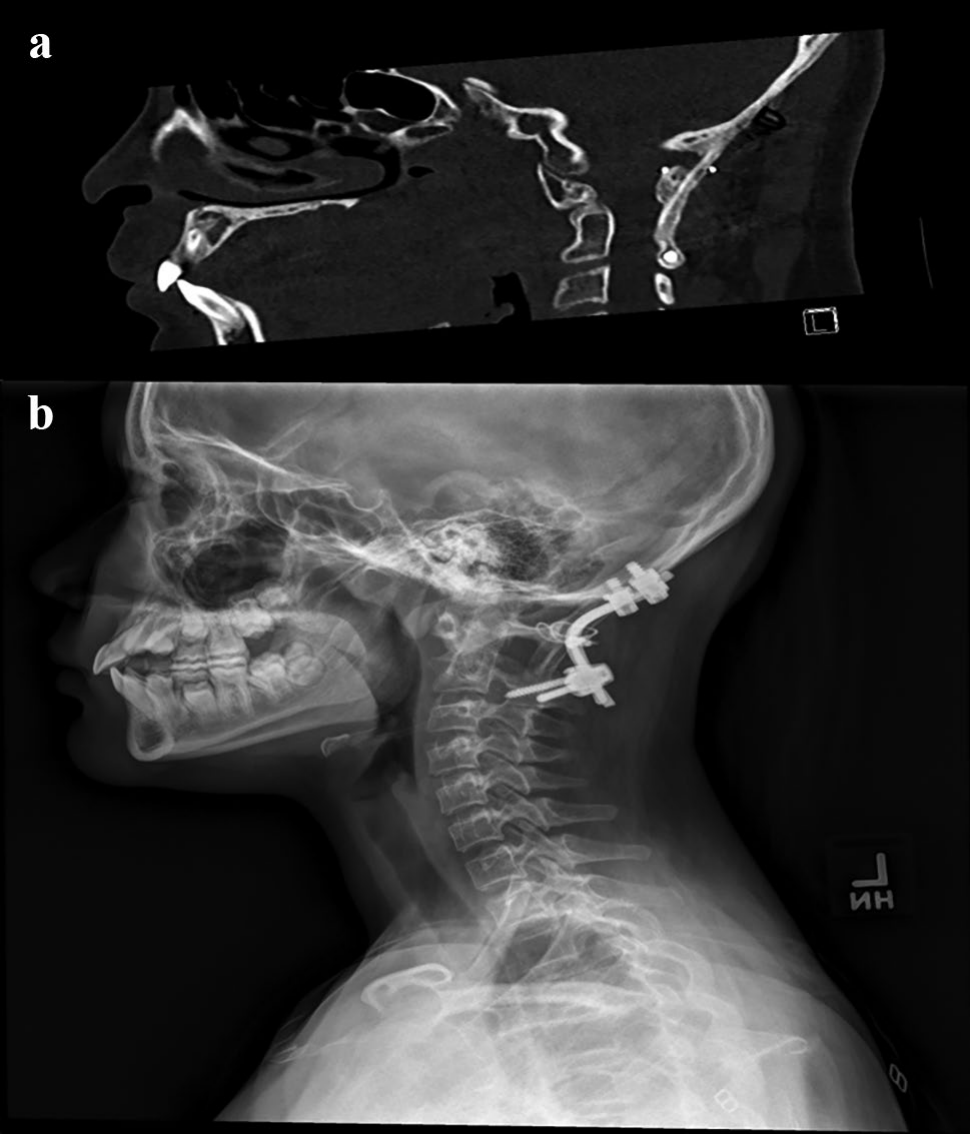
Representative surgical images of fusion construct. **a** Sagittal CT scan showing presence of rib bone graft between the occiput and C2. **b** Representation lateral X-ray obtained postoperatively showing implant construct and sublaminar wiring with rib bone graft

The available surfaces of the occiput, C1 lateral facet, C2 lateral facet, and superior spinous process were then decorticated and structural iliac or rib bone graft obtained and wedged between the occiput and C2 (Fig. 3a). The bone graft was also further held in place by additional sutures or sublaminar wires (Atlas, Medtronic Sofamor Danek, TN) around the hardware. The grafts were overlayed with local autograft bone chips augmented with demineralized bone matrix (DBM), and in some cases, bone morphogenetic protein (BMP) was applied. Where Halo fixation was placed to flip for positioning, the posterior Halo vest and bars were then placed, and the patient flipped supine. Patients without a Halo were placed in a hard cervical collar postoperatively.

### Statistical analysis

Data are presented as means and standard deviations (SD) or proportions for continuous and categorical variables, respectively. Student *t* test and Pearson’s chi-squared test (*X*^*2*^) were used to compare differences in screw lengths relative to age. Statistical significance was assessed at a type I error rate of 0.05. All statistical analyses were performed using the R version 4.0.2 (http://cran.r-project.org/).

## Results

### Demographics and patient pathologies

Twenty-five consecutive pediatric patients underwent O-C fusion incorporating C2 translaminar screw fixation (Table 1). The average age at time of surgery was 10.3 ± 3.9 years. The youngest patient in our series was 2.7 years old. Twelve patients (48%) were female. The average follow-up duration was 42.7 months with a standard deviation of 29.7 months. The most common primary pathology indicated for O-C fusion was 11 patients with compressive os odontoideum.

**Table 1:** Patient characteristics

**Pt**	**Age, gender**	**Condition**	**Instrumentation (plate-rod, or rod with occipital screw ± sublaminar cable), BMP, Halo use, anterior reduction performed**	**f/u (mo)**	**Result**	**Complication**
1*	9, M	Os odontoideum Anterior pannus cord compression	R: C2 TLS 3.5 × 14 mm L: C2 TLS 3.5 × 16 mm C1 sublaminar cables	60	Fused	-
**2**	7, M	AOD trauma	R: C1–C2 transarticular screw L: C2TLS 3.5 × 12 mm	55	Fused	-
3*	16, M	Down AAD	R: C2TLS 3.5 × 20 mm L: C2TLS 3.5 × 18 mm Halo 60 days	37	Fused	Death at 7 months
4*	9, M	Down AAD	R: C2TLS 3.5 × 14 mm L: C2TLS 3.5 × 14 mm Halo 73 days	71	Fused	-
**5**	5, M	AOD trauma	R: C2 TLS 3.5 × 10 mm L: C2 TLS 3.5 × 10 mm	40	Fused	Laminar breech Inadvertent fusion extension to C3
6*	10, F	Skeletal dysplasia (Morquio’s)	R: C2 TLS 3.5 × 14 mm L: C2 TLS 3.5 × 12 BMP	69	Fused	-
**7**	9, F	Os odontoideum, retroflexed dens cord compression with myelomalacia	R: C2 TLS 3.5 × 18 mm L: C2 TLS 3.5 × 18 mm C1 sublaminar cable Transoral odontoidectomy	34	Fused	-
8*	9, M	Down AAD	R: C2 TLS 3.5 × 14 mm L: C2 TLS 3.5 × 16 mm Halo 78 days	59	Nonunion at 12 mo	Nonunion Required revision
**9**	13, F	Chiari 1, basilar impression	R: C2 TLS 3.5 × 18 mm, C3 LMS L: C2 TLS 3.5 × 18 mm, C3 LMS C1 sublaminar cable Anterior reduction performed Bilateral C3 lateral mass screws	46	Fused	Recurrent syrinx, stable junctional kyphosis
10*	8, F	Chiari 1 Basilar impression	R: C2 TLS 3.5 × 16 mm L: C2 LMS Anterior reduction performed	30	Fused	Inadvertent extension of fusion to C3
**11**	14, M	Chiari 1, basilar impression	R: C2 TLS 3.5 × 24 mm L: C2 TLS 3.5 × 22 mm Anterior reduction performed	26	Fused	-
**12**	17, F	Os odontoideum; retroflexed dens cord compression with myelomalacia Shprintzen–Goldberg syndrome	R: C2 TLS 3.5 × 20 mm L: C2 Pars 3.5 × 20 mm Halo 77 days BMP C1 sublaminar cable Transoral odontoidectomy	45	Fused	Breakdown over hardware requiring revision 5 months later
**13**	12, F	Down Os odontoideum retroflexed dens cord compression with myelomalacia	R: C2 TLS 3.5 × 26 mm L: C2 TLS 3.5 × 18 mm Halo 47 days BMP C1 sublaminar cable Transoral odontoidectomy	22	Fused	-
**14**	5, M	AOD trauma	R: C2 TLS 3.5 × 24 mm L: C2 Pars 3.5 × 18 mm	63	Fused	-
**15**	7, M	AOD trauma	R: C2 TLS 3.5 × 18 mm L: C2 TLS 3.5 × 24 mm Halo 118 days C1 sublaminar cable	7	Fused	-
**16**	8, F	Os odontoideum, retroflex dens, cord compression, myelomalacia	R: C2 TLS 3.5 × 18 mm L: None Intraop Halo reduction Halo 84 days BMP C2 sublaminar cable	7	Fused	-
**17**	9, F	Down Os odontoideum retroflexed dens cord compression myelomalacia	R: C2 TLS 3.5 × 10 mm L: C2 TLS 3.5 × 12 mm Intraop Halo reduction Halo 146 days BMP	80	Nonunion at 2 years	Pseudoarthrosis requiring re-fusion 2 years post-op
**18**	15, M	Os odontoideum Retroflexed dens cord compression myelomalacia compression	R: C2 TLS 3.5 × 22 mm L: C2 TLS 3.5 × 20 mm **Transoral odontoidectomy** Preop Halo 7 days Halo 135 days	51	Fused	-
**19**	2, M	Congenital AOD, cord compression C2–C3 kyphotic deformity	L: C2 TLS 3.5 × 16 mm R: None Halo 85 days C1 sublaminar cable Right C3 TLS BMP	42	Fused	-
**20**	14, F	Skeletal dysplasia Os odontoideum retro flexed dens cord compression	R: C2 TLS 3.5 × 16 mm L: C2 TLS 3.5 × 16 mm Intraop halo reduction Halo 100 days BMP	5	Fused	-
**21**	5, F	Chiari 1, basilar invagination brainstem cord compression	R: C2 TLS 3.5 × 18 mm L: C2 TLS 3.5 × 12 mm C1 sublaminar cable Transoral odontoidectomy Halo 132 days BMP	32	Fused	-
**22**	7, M	Os odontoideum, anterior pannus, C1 stenosis cord compression myelomalacia Developmental delay	R: C2 TLS 3.5 × 18 mm L: C2 pars screw Intraop Halo reduction Halo 121 days BMP C1 sublaminar cable	10	Fused	-
**23**	14, F	Os odontoideum Anterior pannus cord compression	R: C2 TLS 3.5 × 22 mm L: C2 TLS 3.5 × 24 mm Halo 99 days, refused C-collar preoperatively BMP C1 sublaminar cable Left C3 lateral mass screw	31	Fused	Had neck pain after head strike after surgery and had course of C-collar
**24**	18, M	Down Os odontoideum (previous nonunion)	R: C2 TLS 3.5 × 16 mm L: C2 TLS 3.5 × 14 mm Halo 84 days	108	Fused	
**25**	11, F	Grisel syndrome AARF	R: C2 TLS 3.5 × 22 mm L: C2 TLS 3.5 × 24 mm Preoperative Halo for reduction Postoperative Halo 17 days BMP C1 sublaminar cable rotatory reduction performed	4	Fused	
	25 patients with 43 total C2 translaminar screws					
Age in years at surgery, mean ± SD	10.3 ± 3.9 Range: 2.7 to 18.6					
Female, *n* (%)	12 (48%)					
Follow-up duration, mean ± SD	42.7 ± 29.7					

### Utilized constructs

**Fig. 4:**
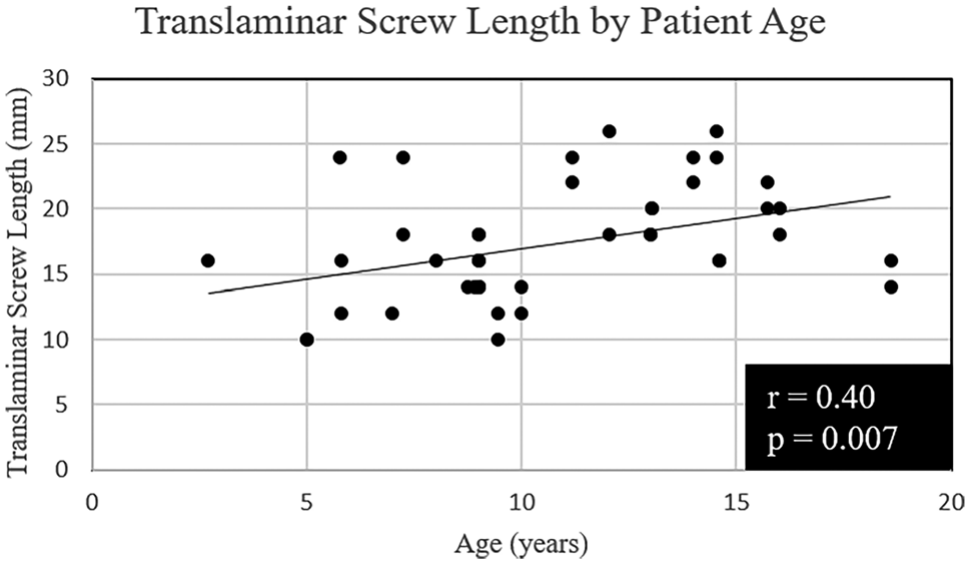
Scatterplot of C2 translaminar screw lengths by patient age. All screws were 3.5 mm in diameter. The Pearson correlation coefficient was 0.40 with a *p* value of 0.007

Of the 25 patients, there were a total of 43 C2 translaminar screws placed, 21 of which were left-sided screws and 22 of which were right-sided screws (Table 2). All patients had at least 1 translaminar screw placed. The screw length ranged from 10 to 26 mm with an average length of 17.1 mm (SD 4.5 mm) on the left and 17.4 mm (SD 4.5 mm) on the right (Fig. 4), which was positively correlated with age (Pearson CC = 0.40, *p* = 0.007). Four patients had hybrid constructs where one patient had an ipsilateral transarticular screw and 3 patients had contralateral pars screws. Two patients had Klippel–Feil deformity of C2 and C3, and the construct was extended to C3 using lateral mass screw. Another patient had a congenital C2–C3 kyphosis, and the construct was extended to C3 using a right C3 translaminar screw. Two patients (8%) had only unilateral translaminar screw with no contralateral screw fixation placed due to unsuitable anatomy for any screw fixation (Table 2).

**Table 2:** Occipitocervical fusion screw placement at C2 characteristics

**Screw type**	**Left C2 screw**	**Right C2 screw**
Translaminar, *n* (%)	21 (84%)	22 (88%)
Screw length (mm), mean (SD)	17.1 (4.5)	17.4 (4.5)
Pars, *n* (%)	2 (8%)	1 (4%)
Transarticular, *n* (%)	0 (0%)	1 (4%)
None, *n* (%)	2 (8%)	1 (4%)

18 (72%) with bilateral C2 translaminar screws

2 (8%) constructs fused to C3 with lateral mass screws and 1 (4%) construct fused to C3 with translaminar screw

**Fig. 5:**
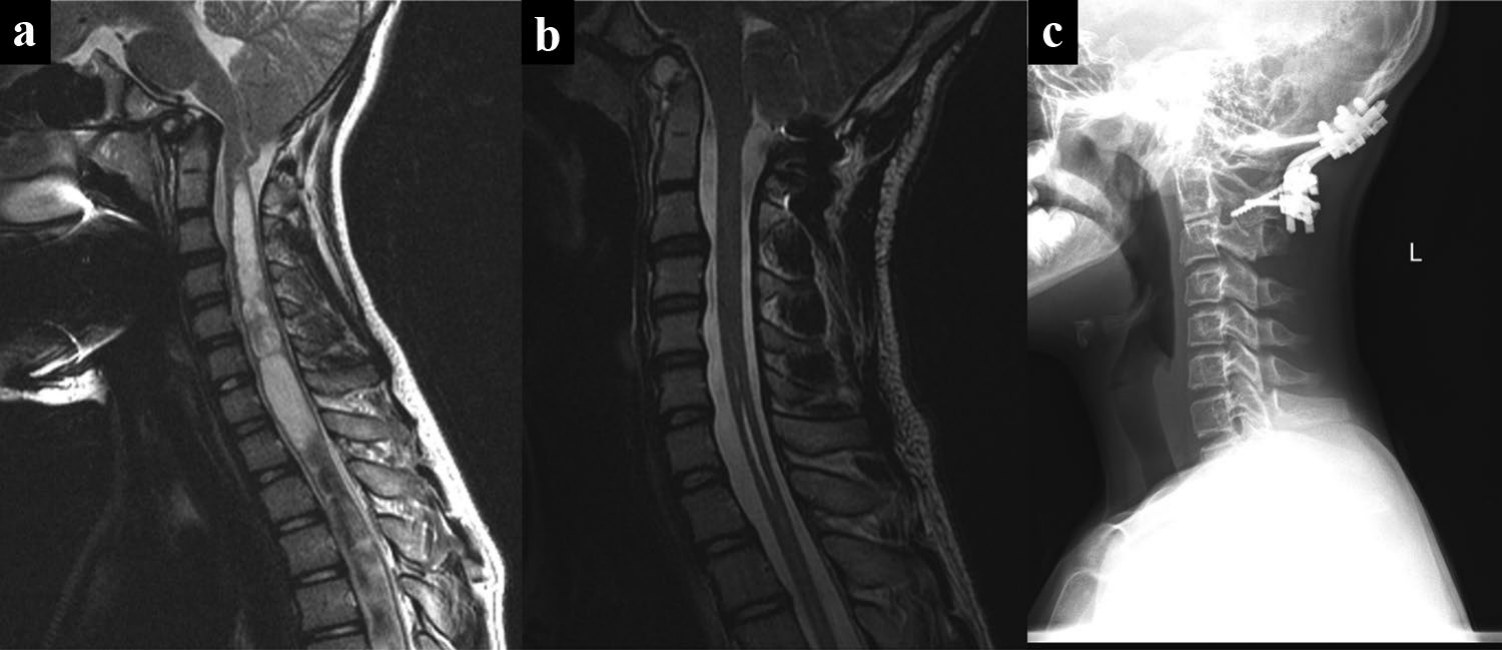
Effects of O-C2 fusion in patients with anterior pathology. **a** Preoperative T2-weighted mid-sagittal MRI demonstrating anterior pathology from basilar invagination and associated cervical cord syrinx. **b** Postoperative T2-weighted mid-sagittal MRI demonstrating reduction of anterior pathology after occipitocervical fusion to C2 utilizing the translaminar screw technique. **c** Lateral cervical spine XR demonstrating hardware with fusion

All patients had either rib and/or iliac crest bone grafts placed between the occiput and C2 which was overlaid with DBM and autologous bone chips (Table 3). Twelve (48%) patients had C1 sublaminar wiring. Eleven (44%) patients had BMP. Sixteen (64%) patients were kept in Halo fixation postoperatively given they were placed in the Halo for the flip or for reduction with the remaining 9 patients (36%) left in hard cervical collars. In addition, 4 (16%) patients had a first stage or concurrent transoral odontoidectomy for basilar impression or irreducible os odontoideum, and another 4 (16%) patients underwent concurrent open reduction of anterior pathology in association with Chiari decompression (Fig. 5).

**Table 3:** Associated surgical adjuncts and interventions

**Surgical adjuncts**	***n* = 25**
Rib and/or iliac graft	25 (100%)
Sublaminar wiring	12 (48%)
Bone morphogenetic protein (BMP)	11 (44%)
Postoperative Halo	16 (64%)
Postoperative C-collar	9 (36%)
**Additional surgical interventions**	
Transoral odontoidectomy	4 (16%)
Anterior reduction performed	4 (16%)

### Outcomes

There were no postoperative neurological complications or injuries (Table 4). One patient had a ventral laminar breech seen on postoperative CT scan which did not require revision. Twenty-three (92%) patients achieved fusion without revision. Two (8%) patients with Down syndrome had a nonunion, one with occipital screw pullout on follow-up. Two (8%) patients had superficial wound dehiscence requiring revision. There was one death 7 months postoperatively after fusion from unknown causes. One patient had junctional kyphosis at C2–C3, which remained stable on follow-up. Two patients (8%) had inadvertent fusion extension to C3.

**Table 4:** Intraoperative complications and postoperative outcomes

**Intraoperative, *n* (%)**	***n* = 25**
Ventral lamina fracture and contralateral screw breech	1 (4%)
Vertebral artery injury	0 (0%)
**Postoperative, *n* (%)**	
New neurological deficit	0 (0%)
Superficial wound dehiscence	2 (8%)
Nonunion requiring reoperation (re-fusion, extension of fusion)	2 (8%)
Adjacent-level kyphosis	1 (4%)
Death of unknown cause	1 (4%)
Inadvertent extension of fusion to C3	2 (8%)

## Discussion

C2 fixation represents a critical anchor point for craniocervical instrumented fusion. Transarticular C1–C2 screws, C2 pars screws, and C2 translaminar screws have been used for C2 screw fixation [14]. Transarticular screws are well-studied and are the most rigid screw fixation method with a 100% rate of O-C fusion reported by Couture et al. [8]. Their insertion risks vertebral artery injury and is not applicable in 11% of pediatric C1–C2 joint spaces due to variations of the vertebral artery course [15, 16]. Vertebral artery injury is also possible with C2 pars screws and can be found in 12.5–20% in an anatomical cadaveric study [17]. C2 translaminar screws have a relatively low risk of vertebral artery injury. Clinical applications in adult series which demonstrate C2 translaminar screws are equally effective as other C2 fixation methods in the upper cervical spine but have a higher rate of pseudoarthrosis and hardware pullout rate in subaxial constructs and longer constructs [18, 19]. In Dorward and Wright’s 7-year series of 52 adult patients with translaminar screw for axis stabilization, the fusion rate was 97.6% with no clinical vertebral artery injury [20]. Complications including dural laceration, dorsal laminar breech, and screw pullout have been reported in adult series [12, 19, 21, 22].

For pediatric application, in addition to the two cases in Wright’s series [11, 13], additional cases of C2 translaminar screw fixation for O-C fusion have been reported in children. Haque et al. placed bilateral C2 translaminar screws in 2 patients, aged 12 and 17, supplemented by rh-BMP-2, for O-C3 fusion [10]. Chamoun et al. utilized translaminar screws in 4 pediatric patients for O-C fusion [23]. Couture et al. placed a translaminar screw in a 1.5-year-old patient attached to a custom loop for O-C fusion, but the screw required removal from laminar breech [8]. Single cases have also been reported or illustrated by Ahmed et al., Bauman et al. and Jea et al. [24–26]. The C2 translaminar screw method in combination with C1–C2 sublaminar wiring has been studied by Keen et al. specifically in a pediatric population with craniocervical dislocation after motor vehicle collision and found that 14 out of 15 patients successfully fused without significant complications and no new neurologic deficits [27]. Hagemann et al. reported on 31 pediatric patients undergoing craniocervical fusion. This study found that fixation with C2 translaminar screws in 19 had a 100% initial fusion rate at 3 months post-op compared to 66.7% in 12 patients without translaminar screw fixation which was statistically significant [28]. The largest pediatric series to date with 39 C2 translaminar screws in 23 patients by Yang et al. when examining all cervical constructs showed no screw-related complications, no neurological injuries, and all patients with clinical union [29]. The use of C2 translaminar screw in pediatric O-C fusion is proposed as the third option in the treatment paradigm proposed by Anderson et al. if C1–C2 transarticular or pars screws are not appropriate, but there has been limited published clinical data [30].

In this series, largest to date in pediatric patients, we demonstrate that C2 translaminar screw is a safe and efficacious strategy when utilized in O-C fusion in the pediatric population. None of the patients in our series required C1 rigid screw fixation, as suggested by Hankinson et al. [31], while 48% had C1 sublaminar cables. O-C fusion with C2 translaminar screws can be achieved without additional subaxial fixation, thereby preserving motion segments. Only 3 patients had additional C3 fixation for specific reasons. The O-C2 translaminar screw construct is also able to reduce anterior pathology when present and reducible and demonstrated an overall first time construct fusion rate of 92%.

**Fig. 6:**
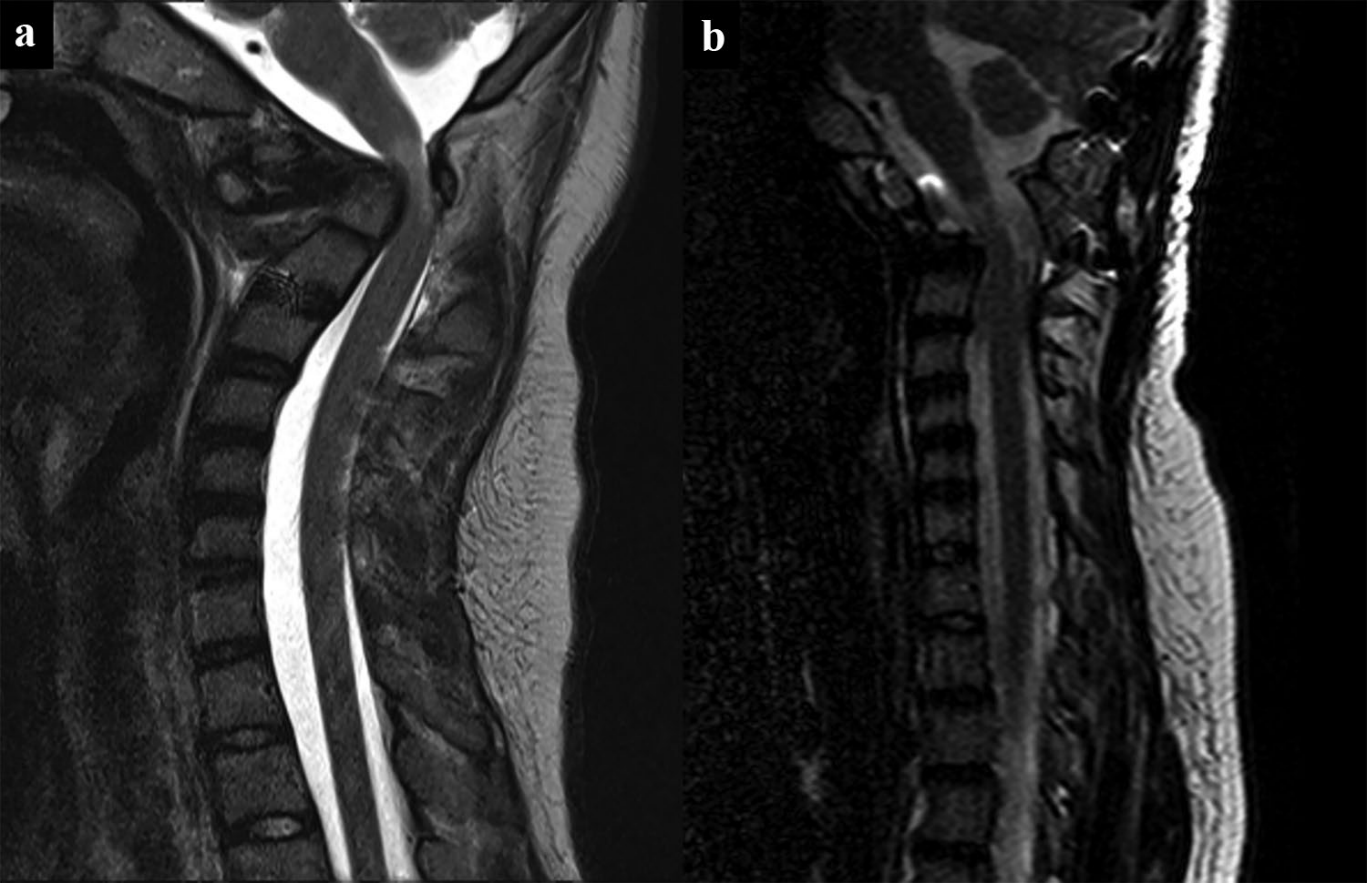
Use of Halo for safe intraoperative flipping. **a** Preoperative sagittal T2-weighted MRI shows compression with myelopathy. This patient was placed in Halo fixation intraoperatively for the flip. **b** Postoperative sagittal T2-weighted MRI 3 years after transoral odontoidectomy and O-C fusion with TLS shows reduction of compression

The use of adjuvant Halo is high is our series and stems from the patient population and our practice of flipping high-risk patients in a halo vest. Many of the patients in this series were highly unstable or had noted cord compression and myelopathy and were placed in a Halo preoperatively for stability or intraoperatively for maximum safety or reduction during the flip to the prone position (Fig. 6). Once the Halo ring was placed with cranial pins, it was felt appropriate to leave the Halo on for postoperative immobilization rather than removing the Halo ring and placing a collar. Additional indications for Halo immobilization were behavior or other concerns of patient compliance for a cervical collar. Previous literature also suggests that Down syndrome patients have lower rates of fusion [32] and all Down syndrome patients were placed in Halos. The two patients in this series with nonunion were patients with Down Syndrome. Both patients had also BMP adjunctively applied illustrating the difficulty in achieving fusion in this population. BMP was also utilized in selected patients in this series where there was a concern regarding the size and quality of the donor graft. Inadvertent fusion extension is a known complication of pediatric spine surgery. Meticulous soft tissue dissection to avoid exposure of adjacent bony surface is required.

**Fig. 7:**
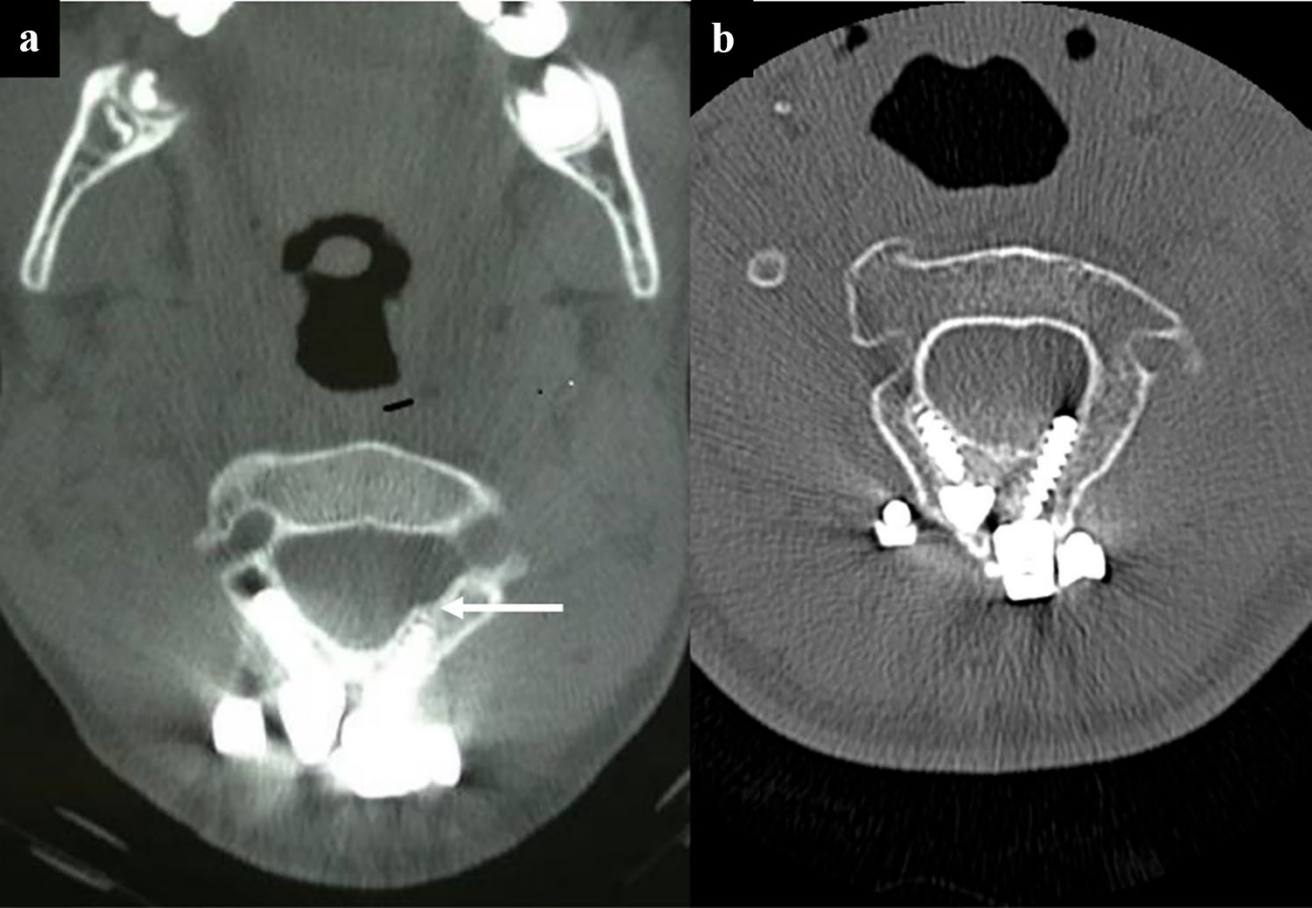
Reduction of surface area for fusion and ventral breach of translaminar screws. **a** This axial CT scan of the C2 laminae with implanted hardware demonstrates the reduced surface area available for bony fusion due to the presence of the screw heads and the offsets that are necessary for attachment of the translaminar screws to the rod. Of note, this CT scan also demonstrates laminar breech of the left translaminar screw with a fractured cortical shell (white arrow). **b** This axial CT scan of the same patient taken 7.5 years later to follow-up on the ventral fracture demonstrates that cortical covering of the breech has remodeled and the screw threads protrudes more in the canal without a cortical layer

In this series, the minimum dimension of C2 lamina thickness was chosen as 4 mm for 3.5 mm diameter translaminar screws. In Chern’s study, 88.4% of children between 1.5 and 16 have a laminal thickness greater or equal to 4 mm, so most children can accommodate at least one translaminar screw. The technique of bilateral screw insertion in smaller C2 spinous processes that cannot accommodate crossing screws is achieved by partially resecting the spinous process and placing translaminar screws on each side at the dorsal aspect of the lamina, and the sensible limit of bilateral C2 translaminar screw placement is exceeded when there is sufficient hardware crowding of the screw heads at the dorsal aspects of the C2 to displace the placement of second translaminar screws to a more ventral direction resulting in ventral fracture or ventral laminar or foraminal breech (Fig. 7a). In our patient with ventral breech, subsequent imaging 7.5 years later to follow-up on the ventral fracture demonstrated that cortical covering of the breech had remodeled and the screw threads protruded more in the canal without a cortical layer (Fig. 7b). This has not been previously reported and illustrates the unique considerations of instrumenting the pediatric spine. In our construct, offsets were used for fixation of the rod to the more medial C2 translaminar screw head position. The offsets, along with the polyaxial screw heads, significantly reduced the surface for fusion by covering the dorsal lamina of C2, particularly in pediatric patients with less bony surface area (Fig. 7). To achieve fusion, we used a structural posterior iliac or rib graft meticulously wedged between the caudal aspect of C2 to the occiput often tied down with additional sutures or cables to the hardware. This was supplemented by morselized bone matrix grafting lateral to the rod between the C2 facet and occiput.

## Conclusion

Translaminar screw fixation of C2 is an effective option in pediatric O-C fusion. The ability to place translaminar screw fixation in children is limited by the size and growth considerations of pediatric C2. The choice amongst surgical techniques for O-C fusion in children depends on the particular anatomy and knowledge of the limitations of each fixation technique in order to achieve the highest rate of fusion with the lowest risk. When the anatomy is suitable, C2 TLS may be chosen as an option in pediatric patients requiring O-C fusion.
